# Protective Immunity Induced by an *Eimeria tenella* Whole Sporozoite Vaccine Elicits Specific B-Cell Antigens

**DOI:** 10.3390/ani11051344

**Published:** 2021-05-09

**Authors:** Marco A. Juárez-Estrada, Amanda Gayosso-Vázquez, Guillermo Tellez-Isaias, Rogelio A. Alonso-Morales

**Affiliations:** 1 Departamento de Medicina y Zootecnia de Aves, Facultad de Medicina Veterinaria y Zootecnia, Universidad Nacional Autónoma de México, Coyoacán, Cd. De México 04510, Mexico; 2 Departamento de Genética y Bioestadística, Facultad de Medicina Veterinaria y Zootecnia, Universidad Nacional Autónoma de México, Coyoacán, Cd. De México 04510, Mexico; amandagv66@hotmail.com (A.G.-V.); ralonsom@unam.mx (R.A.A.-M.); 3 Department of Poultry Science, University of Arkansas, Fayetteville, AR 72701, USA; gtellez@uark.edu

**Keywords:** coccidiosis, immunogen, immunodominant, nanoparticle adjuvant, merozoite, crowding dose

## Abstract

**Simple Summary:**

Coccidiosis caused by *Eimeria tenella* is a dreadful disease with a significant economic impact to the poultry industry. The disease has been controlled by routine medication of feed with synthetic chemicals or ionophore drugs. However, the rising appearance of drug resistance and public demands for reduced drug use in poultry production have driven a dramatic change, replacing anticoccidial drugs with alternative methods, such as vaccination with either virulent or attenuated *Eimeria* oocysts. Based on preliminary studies, the immune protection evaluating whole-sporozoites of *E. tenella* vaccine was verified. After this vaccine provided successful protection, the humoral response of a heterologous species like the rabbit was compared with the natural host immune response. Several B-cells antigens from the *E. tenella* sporozoite suitable for a genetically engineered vaccine were identified. Vaccination with newly identified recombinant antigens offers a feasible alternative for the control of avian coccidiosis into the broiler barns favoring the gradual withdrawal of the anticoccidial drugs.

**Abstract:**

This study investigated protection against *Eimeria tenella* following the vaccination of chicks with 5.3 × 10^6^ *E. tenella* whole-sporozoites emulsified in the nanoparticle adjuvant IMS 1313 N VG Montanide™ (EtSz-IMS1313). One-day-old specific pathogen-free (SPF) chicks were subcutaneously injected in the neck with EtSz-IMS1313 on the 1st and 10th days of age. Acquired immunity was assayed through a challenge with 3 × 10^4^ homologous sporulated oocysts at 21 days of age. The anticoccidial index (ACI) calculated for every group showed the effectiveness of EtSz-IMS1313 as a vaccine with an ACI of 186; the mock-injected control showed an ACI of 18 and the unimmunized, challenged control showed an ACI of −28. In a comparison assay, antibodies from rabbits and SPF birds immunized with EtSz-IMS1313 recognized almost the same polypeptides in the blotting of *E. tenella* sporozoites and merozoites. However, rabbit antisera showed the clearest recognition pattern. Polypeptides of 120, 105, 94, 70, 38, and 19 kDa from both *E. tenella* life cycle stages were the most strongly recognized by both animal species. The *E. tenella* zoite-specific IgG antibodies from the rabbits demonstrated the feasibility for successful B cell antigen identification.

## 1. Introduction

Coccidiosis is a major parasitic disease that has a great economic impact on poultry production. The average cost of coccidiosis per chicken produced is GBP 0.16 globally [1]. *Eimeria tenella* is probably the most important species of *Eimeria* that infects chickens; it is the causative agent of cecal coccidiosis, causing tissue damage that results in blood loss, dehydration, nutrient malabsorption, and increased susceptibility to other opportunistic pathogens [1,2]. A conventional control strategy is achieved by biosecurity measures combined with in-feed anticoccidial drugs or vaccination with live virulent or attenuated parasites [1,2]. However, alternative control strategies are urgently needed due to the possible upcoming bans restricting the use of anticoccidials as feed additives [1,3]. The development of subunit and recombinant vector vaccines is currently being pursued. Progress has been made toward the development of these vaccines using *E. tenella* antigens such as apical membrane antigen 1 (AMA1), immune mapped protein 1 (IMP1), microneme protein 1 (MIC1), microneme protein 2 (MIC2), microneme protein 3 (MIC3), SO7, rhomboid-like protein, profilin (3-1E), and TA4; however, many immune challenge reports have shown only partial protection [3,4,5]. Vaccines with fractioned proteins of the *E. tenella* sporozoite have been used to test immunoprotection in challenge trials. Almost all of them have been shown to decrease the oocyst shedding of birds significantly [6,7,8,9,10,11,12]. The sporozoite stage plays a critical role in invasion and likely represents the most amenable target for the host immune response, since in immune birds, they undergo a very restricted development or even fail to penetrate cells in the intestinal tract [8,13,14]. The surface antigens are present on the sporozoite and some of these molecules have roles in host–parasite interactions, probably because these are naturally exposed during parasite recognition and invasion of the intestinal cells [8,14,15,16]. Until now, it has been difficult to find a single target antigen that can elicit sterilizing immunity against coccidiosis [3,4,13]. Therefore, a mixture of antigens, which can act synergistically, might reduce oocyst output. The screening of sporozoites that highly express secreted antigens or surface antigens is crucial for the development of anticoccidial vaccines [5,14]. Immune responses to *Eimeria* involve many facets of innate and adaptive/acquired immunity, the latter encompassing both cellular and humoral immune mechanisms [2,9,17,18]. T-cells play a critical role in protective immunity against *Eimeria* infections; based on the comprehensive consideration of this aspect, researchers commonly integrate only T-cell epitopes in their DNA, subunit, and recombinant vaccines [3,4]. However, it has been demonstrated that class B antigens are present in all seven species of chicken coccidia, indicating that this class of antigen could protect chickens against coccidiosis [13,19,20]. Therefore, in order to induce a successful cellular immune response, an effective synthetic vaccine against coccidiosis should contain a greater amount of both protective T- and B-cell antigens [3,4,18,21]. The current study was undertaken to evaluate whole sporozoites of *E. tenella* emulsioned in a novel nanoparticle adjuvant (IMS 1313 N VG Montanide™) as a potential vaccine against avian coccidiosis. In order to identify B-cell antigens involved in the immune response, this same vaccine was also subcutaneously injected into rabbits and naïve birds. Antisera from these animals were probed to identify specific highly immunogenic antigens of two asexual zoite stages of *E. tenella*. If, with time, the immunogenicity of subunit vaccines can be improved by incorporating appropriate T- and B-cell antigens, these vaccines could represent the next generation of highly efficient anticoccidial strategies.

## 2. Materials and Methods

### 2.1. Parasites

The wild-type strain of chicken *Eimeria* used in these experiments was originally isolated from birds with clinical signs of cecal coccidia in a broiler farm in Mexico. The *E. tenella* strain used is the progeny of a single oocyst. Oocysts were separated from feces, sporulated, and stored as described previously [22]. Methods for preparing infective doses, and quantifying infections by counting the oocysts per gram of feces (OPG) were followed as previously described by Juárez et al. [22]. In order to maintain viability, parasites were passaged at frequent intervals using 3-week-old specific pathogen-free (SPF) White Leghorn chicks and routinely used within 30 days after sporulation.

### 2.2. Animals

For the immunization challenge trial, eighty one-day-old SPF White Leghorn chicks were individually weighed and randomly assigned to 8 cages (10 birds per cage) in a battery brooder housed in an environmentally controlled room under specific pathogen-free conditions. All chicks were fed with coccidiostat-free feed and water ad libitum. In addition, two White New Zealand rabbits and two SPF White Leghorn chickens used in the experimental immunization assay were kept in an adjacent isolation room. Standard biosecurity management was implemented to avoid any unusual exposure of chickens or rabbits to coccidia during the experimental immunization/challenge test.

### 2.3. Purification of Sporocysts and Sporozoites from E. tenella Oocysts

The release of sporocysts was achieved by vortexing sporulated oocysts (2.5 × 10^7^/mL) at 2000 rpm with 1 mm-diameter glass beads (Sigma, St. Louis, MO, USA) for 70 s. Sporocysts were purified using a 50% Percoll gradient (density: 1.13 g/mL, GE Healthcare, Piscataway, NJ, USA), resuspended at 1 × 10^6^/mL in excystation medium, and incubated at 42 °C for 150 min. Freshly excysted sporozoites were washed and purified on a 60% Percoll gradient (density: 1.13 g/mL, GE Healthcare, Piscataway, NJ, USA) [23]. Sporozoites for the immunization schedule were resuspended in sterile PBS, gradually frozen at −70 °C at a rate of 1 °C/min, and stored at −70 °C until use.

### 2.4. Preparation of the E. tenella Whole-Sporozoite Vaccine

The *E. tenella* sporozoites previously inactivated by freezing were thawed and resuspended in sterilized PBS, then centrifuged for 10 min at 2000 × *g*, 4 °C. The pellet was collected and resuspended in sterilized PBS and counted in a Neubauer counting chamber, as described elsewhere [22]. Sporozoite density was adjusted to 1.06 × 10^4^ sporozoites/µL of PBS. The immunogen was prepared by emulsifying the sporozoites (5.3 × 10^6^ sporozoites/0.5 mL of PBS) with 0.5 mL of IMS 1313 N VG nanoparticle adjuvant (Montanide™, Seppic, Paris, France) (1 mL = 1 dose) following the manufacturer’s instructions.

### 2.5. Preparation of Antigens from Sporozoites and Merozoites

Sporozoites (Sz) were obtained and purified as previously described by Dulski and Turner [23]. Second generation merozoites (Mz) were purified using the technique described by Liu et al. [24]. Parasites for antigen preparation were resuspended in sterile PBS with protease inhibitor (cOmplete™ Roche Applied Science, Mannheim, Germany). Sporozoites and merozoites were separately disrupted by 5 freeze–thaw cycles. The final suspension was centrifuged at 2000 × *g* for 18 min at 4 °C. The supernatants were collected, and the protein concentration was estimated by Bradford assay following the manufacturer’s instructions (BioRad, Hercules, CA, USA). Antigens were stored in 500 μL aliquots at −70 °C until use.

### 2.6. ELISA

Indirect ELISA assay was used to test the reactivity of antisera to sporozoite and merozoite antigens, as described by Constantinoiu et al. [25]. Antisera from birds who had been immunized and challenged and antisera from the rabbits and SPF chickens vaccinated to the immunization schedule were assayed separately. Serum samples from previously non-immunized SPF White Leghorn chicks were included as controls in every plate. The positive reference antisera were previously obtained from three SPF White Leghorn chicks vaccinated subcutaneously four times (interval: two weeks) with 100 μg of complete *E. tenella* sporozoites per bird. All sera samples were diluted 1:100 and tested in duplicate as previously described Constantinoiu et al. [25]. All samples were analyzed three independent times to identify outliers.

### 2.7. Western Blotting

Twenty micrograms of each purified fraction of *E. tenella* asexual zoite stages were separated by 12% reducing SDS-PAGE, electrotransferred onto 0.2 µm pore-size nitrocellulose membrane (NCM) (Bio-Rad, Hercules, CA, USA), and probed with the antisporozoite *E. tenella* serums from immunized rabbits and birds, preimmune sera and antisera at 14th days were diluted at 1:100 (*v*/*v*), at 28th 1:200, and at 49th days post-immunization samples of rabbits were diluted 1:8000 and birds 1:2000. Horseradish peroxidase conjugate (HRP) (anti-rabbit, anti-chicken) (Jackson ImmunoResearch Laboratories, Inc. West Grove, PA, USA) was used as a secondary antibody at a dilution of 1:2000 for rabbit IgG, and at 1:1500 for chicken IgY. The blots were visualized with 3,3′-diaminobenzidine (10 mg/5 mL) (DAB tablets, SigmaFast™, Sigma-Aldrich Corp., St. Louis, MO, USA).

### 2.8. Antisporozoite Immunization in Rabbits and SPF Birds

Two New Zealand White (NZW) rabbits (2 months of age) and two SPF White Leghorn chickens (120 days of age) were immunized with the EtSz-IMS1313 vaccine multiple times. The primary immunization in both species was given at the same time. The immunization was boosted every two weeks until 42 days after the first immunization (PI) using the same dose each time. In rabbits, the entire dose (1 mL) was administered subcutaneously in multiple sites on a shaven patch of the back. In SPF birds, the entire dose (1 mL) was administered subcutaneously in the neck dorsally and posterior to the head.

### 2.9. Oocysts and Serum Samples

Droppings from every group were collected for 3 days, starting on the 5th day and finishing on the 7th day post-challenge (PC). Oocyst output was calculated as the OPG shedding from every group. Control birds were checked for OPG throughout the whole experiment. At three days of age, three serological samples were pooled from the sera of three naïve birds. Five birds from each group of the immunized challenge test were bled at 21 days of age and the serological samples were pooled. The NZW rabbits and SPF birds from the immunization schedule were bled on the 1st, 14th, 28th, and 49th day post-immunization. Sera were separated by centrifugation at 2000 × *g* for 5 min and stored at −20 °C until use. Serological samples were individually analyzed.

### 2.10. Experimental Design

Chickens were caged in four groups of ten (two replicates per treatment). Throughout the whole experiment, group 1 was left as unimmunized and unchallenged control birds. In group 2, the entire dose (1 mL) of the sporozoite vaccine was injected subcutaneously (SC) in the neck dorsally and posterior to the head of one-day-old chicks. The 20-gauge 1-inch needle was inserted caudally, parallel to the cervical vertebrae, for at least 1.2 cm to deposit the antigen distant to the injection site to prevent vaccine seepage. A booster dose was given at 10 days of age. Birds in group 3 were mock-injected with 1 mL of adjuvant emulsified in sterilized PBS under the same schedule applied in group 2. Chicks in group 4 (sham inoculated) were only injected with 1 mL of sterile PBS. At 21 days of age, every bird from groups 2, 3, and 4 were challenged per os with 3 × 10^4^ *E. tenella* sporulated oocysts. Naïve birds from group 1 received only distilled water by oral gavage. At 21 days of age and 7 days PC, all birds were individually weighed. On day 28, all birds were euthanized, and lesion scores were assayed. During the prepatent period, mortality was recorded daily.

### 2.11. Parameters to Assess Immune Protection

The efficacy of vaccination with EtSz-IMS1313 was evaluated on the basis of survival rate, body weight gain, oocyst output decrease ratio, lesion score, and anticoccidial index (ACI). Survival rate was estimated by the number of surviving chickens divided by the number of initial chickens ×100. Average body weight gain (g) was assessed at 21 days of age (before challenge) and the relative body weight gain rate (%) was assessed at 28 d (7 d PC). In brief, body weight gain was calculated as follows: the body weight at 28 d minus the body weight at 21 d. The relative body weight gain was calculated as follows: the average body weight gain of chicks in the vaccinated group or challenged control group/the average body weight gain of chickens in the unchallenged control group ×100. The OPG for each group was counted using the McMaster counting technique, as described by Juárez et al. [22]. The oocyst output decrease ratio (%) was calculated as follows: the number of oocysts from challenged control birds minus the number of oocysts from immunized birds/the number of oocysts from challenge control birds ×100. Reductions in the percentage in oocyst shedding of 65% or greater were considered protective [26]. At 7 days PC, cecal lesions (all birds per group) from each group were scored on a graded scale from 0 (normal) to 4 (severe) in a blinded fashion by two independent examiners following the method described by Johnson and Reid [27]. The lesion score protective index (LSPI) was calculated as follows: [1 − (IC/NIC)] × 100, where IC = the mean lesion score for the immunized/challenged group and NIC = the mean lesion score for the sham-inoculated/challenged group. Reductions in lesions scores of 65% or greater were considered protective. ACI is a synthetic criterion for assessing the protective effect of a medicine or vaccine and is calculated as follows: (survival rate + percent relative weight gain) − (lesion index + oocyst index) [28].

All data were subjected to analysis of variance (ANOVA) as a completely randomized design, using the general linear model procedure of SAS/STAT 9.1 data editor software (SAS Institute Inc., Cary, NC, USA). Significant differences among the means were determined by Tukey’s multiple range test at *p* < 0.05. All data were expressed as the mean ± SD of all birds per group. Predominant bands identified on the Western blot of both life cycle stages of *E. tenella* were graded by three independent observers in order to identify quirks.

### 2.12. Ethical Approval

The use of animals for the experimental studies applied here was approved by the Board for Husbandry and Care in Animals of the Faculty of Veterinary Medicine and Zootechnic U.N.A.M. Mexico through the PhD internal board approval number: 1239522578/2018-1.

## 3. Results

As a consequence of the challenge, almost a third of the chicks in the mock-immunized (adjuvant) and unimmunized groups died. No deaths occurred in the other experimental groups. Weight gains were assessed at day 28 (7 d PC); compared to the unimmunized unchallenged control group, the relative body weight gain in the sporozoite-vaccinated group was 94.8%, which was significantly higher than that in the mock-immunized challenged chickens (23.0%) and the unimmunized challenged chickens (−27.2%, Table 1). The average cecal lesion scores in the unimmunized birds, mock immunized control, and chickens immunized with intact sporozoites of *E. tenella* were recorded as +3.80 ± 0.25, +3.03 ± 0.79, and 0.69 ± 0.70, respectively (Table 1). The lesion score protective index of the chickens immunized with the whole sporozoites of *E. tenella* (81.78%) was substantially higher compared with the LSPI of the mock-immunized-challenged chickens (20.34%) or the LSPI of the unimmunized challenged chickens (0.0%, Table 1). The oocysts output decrease ratio of the vaccinated challenged chickens was significantly reduced (95.8%) compared with the shedding oocysts of the unimmunized challenged chickens (Table 1). The anticoccidial index showed the effectiveness of the sporozoite vaccine (ACI = 186) in comparison with the unimmunized challenged chicks (ACI = −28) or the mock-immunized challenged chicks (ACI = 18, Table 1).

**Figure 1:**
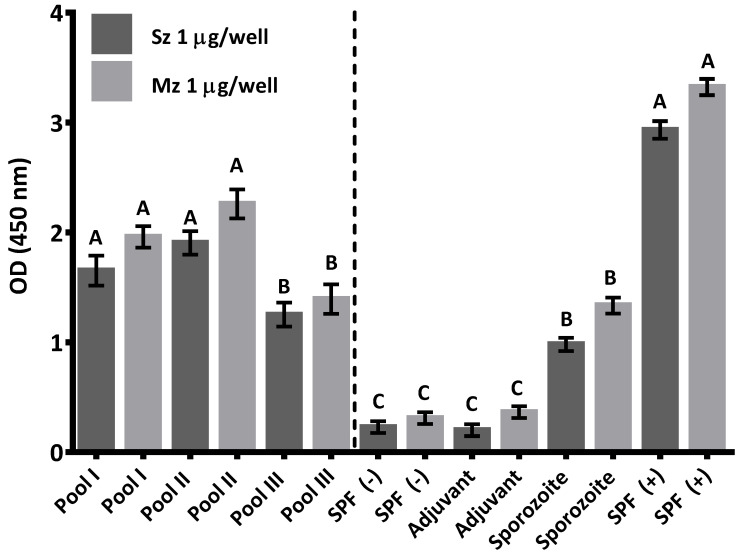
Mean avidity of sera against sporozoite (Sz) and merozoite (Mz) antigens in SPF chicks at 3 days of age (Pool I, II, and III) and antibody response at 21 days of age in the chicks immunized with whole sporozoites of *E. tenella*, chicks mock-injected, unimmunized SPF birds (−), and positive reference sera (+). Bars represent mean OD (± SD). Different letters (A, B, or C) in sporozoite or merozoite antigens of *E. tenella* indicate statistical differences at *p* < 0.05.

At 3 days of age, the humoral response of naïve SPF chicks to both life cycle stages of *E. tenella* was evaluated (Figure 1). All sera pools from naïve SPF chicks showed a high immune reactivity against both *E. tenella* asexual zoite stages; however, this response was slightly higher towards merozoites than sporozoites (Figure 1). On the other hand, at 21 days of age, the antibody titer of *E. tenella* from the group immunized with whole sporozoites of *E. tenella* was significantly higher (*p* < 0.05) than the antibody level of mock immunized and unimmunized birds (Figure 1).

**Figure 2:**
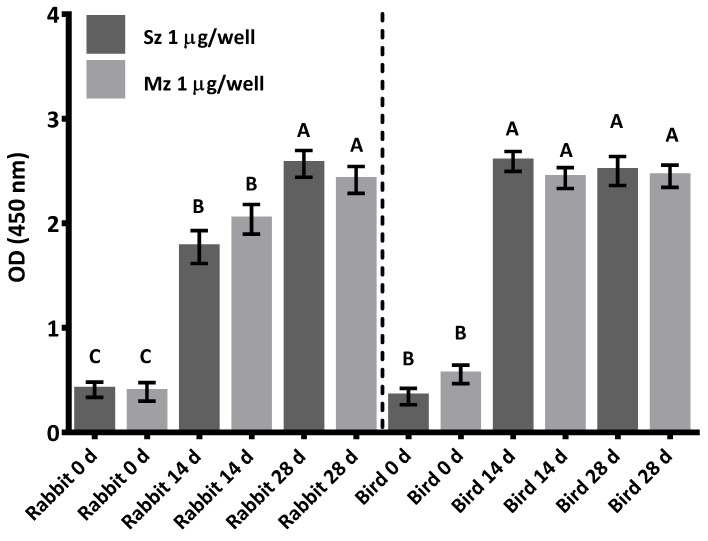
Dynamics of antibody production against two life cycle stages of *E. tenella* (Sz and Mz) in rabbits and SPF birds at 0, 2, and 4 weeks after immunization with whole sporozoites of *E. tenella*. Preimmune sera (0 d) were collected prior to the first immunization. The rabbit and SPF chicken antisera were diluted 1:100. Bars represent mean values with respective SDs. Different letters on same type of antigen indicate statistical differences at *p* < 0.05.

Figure 2 shows the dynamics of the antibody response to sporozoite and merozoite antigens in rabbits and SPF birds immunized with whole sporozoites of *E. tenella*. Antibody production was similar in rabbits and chickens; however, chicken titers reached a plateau after the second week, while the rabbits did not reach this plateau until the fourth week (*p* < 0.05). The rabbits and SPF birds produced cross-reacting antibodies either to sporozoites or merozoites in a very similar pattern. Although immunization was performed with sporozoites, the antibody responses in both species demonstrated a similar reactivity toward merozoites (Figure 2).

**Figure 3:**
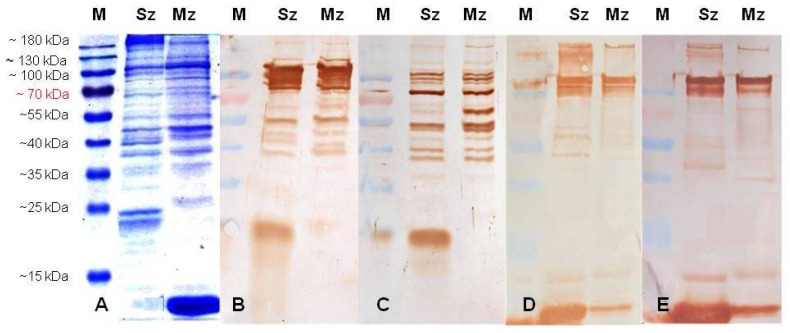
Proteins from the sporozoite (Sz) and the 2nd generation of the merozoite (Mz) of the wild-type *E. tenella* strain resolved by 12% SDS-PAGE and stained with Coomassie Brilliant Blue (**A**) or probed in immunoblots with antisera (1:100) from two rabbits (**B**,**C**) and two SPF birds (**D**,**E**) at 14 days post-immunization with 5.3 × 10^6^ whole sporozoites of *E. tenella*. M: molecular weight ladder in kDa.

**Figure 4:**
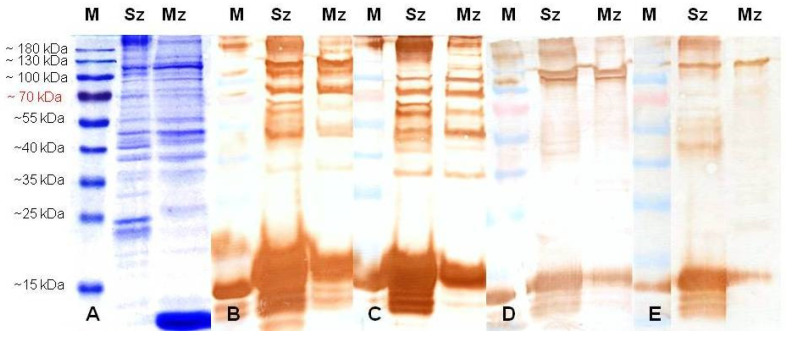
Proteins from the sporozoites (Sz) and merozoites (Mz) of the wild-type *E. tenella* strain resolved by 12% SDS-PAGE and stained with Coomassie Brilliant Blue (**A**) or probed in immunoblots with antisera (1:200) from two rabbits (**B**,**C**) and two SPF birds (**D**,**E**) at 28 days post-immunization with 5.3 × 10^6^ whole sporozoites of *E. tenella*. M: molecular weight ladder in kDa.

**Figure 5:**
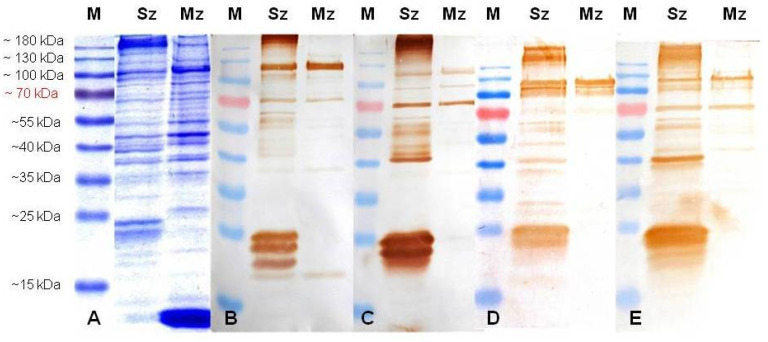
Proteins from the sporozoites (Sz) and merozoites (Mz) of the wild-type *E. tenella* strain resolved by 12% SDS-PAGE and stained with Coomassie Brilliant Blue (**A**) or probed in immunoblots with antisera from two rabbits (diluted at1:8000) (**B**,**C**), and two SPF birds (diluted at 1:2000) (**D**,**E**) at 49 days after four immunizations with 5.3 × 10^6^ whole sporozoites of *E. tenella*. M: molecular weight ladder in kDa.

The *E. tenella* sporozoite proteins analyzed by 12% SDS-PAGE showed relevant bands at about 185, 105, 94, 68, 47, 40, and 38 kDa and two bands close to 25 kDa, while merozoites showed preponderant bands at 105, 82, 55, 47, 38, 26, and 12 kDa (Figure 3A, Figure 4A, and Figure 5A).

Polypeptides from the two life cycle stages of *E. tenella* were blotted onto NCM and probed with a panel of antibodies raised in rabbits and SPF birds against the whole sporozoites of *E. tenella*. Sporozoites contain many polypeptides that are recognized by the antisera of both species, and merozoites also contain several polypeptides that cross-react with these same antibodies (Figure 3). The antibodies raised to one specific stage recognized conserved epitopes on antigens from another stage. At 14 days PI, the antisporozoite sera from the rabbits (Figure 3B,C) recognized a wider antigenic pattern in the sporozoite and merozoite antigens than the antisera from the SPF birds (Figure 3D,E). Both species recognized antigens with a molecular weight greater than 50 kDa, among which 120, 105, 94, 82, and 55 kDa polypeptides in both *E. tenella* asexual zoite stages are relevant (Figure 3). Preimmune serum from rabbits and SPF birds maintained separately under *Eimeria*-free conditions showed no antigen recognition in both *E. tenella* asexual stages (data not shown).

When excess antibodies were used for probe blots (e.g., antisporozoite sera diluted 1:200 at 28 days PI, Figure 4), more polypeptides were reactive to rabbit antisera, but the background was higher than that observed using the antisera of both SPF birds. Although after four boosters with whole sporozoites of *E. tenella*, the rabbits and SPF chicks responded almost the same to the *Eimeria* antigens, the rabbits showed a stronger antibody response than the chickens. In fact, in order to optimize the signal to noise ratio for the Western blot, both rabbit antiserums had to be diluted 1/8000 (Figure 5B,C); instead, SPF chicken antisera were only diluted 1/2000 (Figure 5D,E). In this way, at 49 days PI, the sporozoites showed major bands at about 120, 105, 94, 70, 64, 58, 55, 47, 44, 42, 38, 24, 23, 21, and 19 kDa, and the merozoites at 120, 105, 70, 38, and 19 kDa (Figure 5). Remarkably, all antibodies against the sporozoite recognized four reduced polypeptides from 19 to 24 kDa in the sporozoite, and a doublet at around 70, 105, and 120 kDa in the sporozoites and 2nd generation of merozoites (Figure 5).

## 4. Discussion

Poultry coccidiosis affects welfare and costs of poultry production. While live coccidial vaccines is limited by their low reproductive index and high production costs, at this time is the only reliable commercial tool available as an alternative to anticoccidial drugs [3,4,29]. In recent years, several studies have shown that recombinant vaccines in combination with novel adjuvants may offer a stronger mucosal immunity that stimulates both innate and acquired (humoral and cell-mediated) immune responses against several *Eimeria* strains [30,31,32,33,34,35]. The delivery system of recombinant or subunit vaccine candidates, using eukaryote or prokaryote vectors for feed or water administration application have shown optimistic and realistic results [36,37,38,39,40]. Some of these novel vaccines not only provide antigenic diversity, but cost of production and welfare issues are significantly reduced or eliminated [41,42,43].

The identification of immunogenic epitopes specific to parasite life cycle stages conveying protective immunity is a pivotal step in recombinant vaccine development [3,4,5,14,19]. In this study, an immunological approach was used to define the immune protection and antigenic polypeptide repertoire in SPF chicks and rabbits immunized with whole sporozoites of *E. tenella*. Yun et al. [18] stated that some killed parasites administered as vaccines by different routes failed to induce protective immunity. Nonetheless, numerous studies have been carried out worldwide using soluble proteins from the sporozoite of *E. tenella* as a vaccine, and this type of immunogen has induced immune protection between 30% and 70% against homologous *E. tenella* challenge [6,7,8,9,10,11,12]. To our knowledge, there are no previous reports on the use of the whole inactivated sporozoites as a vaccine in homologous challenge trials.

Lesion score, oocyst output, and body weight gain are the main criteria of the disease burden of chicken coccidiosis [28]. Hence reducing the disease burden has practical significance in poultry production and is regarded as the evaluation criteria for the evaluation of vaccine efficacy and protective immunity in avian coccidiosis [26,28]. Our results revealed that the birds injected subcutaneously with whole sporozoites of *E. tenella* emulsified in Montanide™ IMS 1313 N VG were successfully protected against challenge with a heavy dose of *E. tenella* sporulated oocysts. An important component of our vaccine was the adjuvant, the nanoparticle-based adjuvant IMS 1313 N VG administered s.c. was successful in potentiating the immunogenicity and immune protection against whole sporozoites of *E. tenella.* To the best of our knowledge, this adjuvant has never been used with whole sporozoites of *E. tenella* in any immunization/challenge experimental trial of clinical coccidiosis. Therefore, further studies are needed to uncover the major antiprotozoal properties mediated by this nanoparticle-based adjuvant in chicken *Eimeria* infections.

Numerous studies have been carried out worldwide to elucidate the mechanism of protective immunity against coccidiosis [2,6,9,12,17,18,20]. It was believed from early studies that cellular immunity plays the most important role in the protection against *Eimeria* sp., whereas humoral immunity plays a minor role in resistance against infection [2,17,18]. However, recent results showed the ability of antibodies raised by live immunization or against some specific life cycle stages of *Eimeria* sp. to inhibit parasite development in vitro and in vivo and demonstrated the role of antibodies in the protection against avian coccidiosis [20].

To evaluate the immune response to the EtSz-IMS1313 vaccine in chickens, we also measured the antibody levels and antigen recognition in two asexual zoite stages of *E. tenella*. At 21 days of age, there was no significant antibody response in mock-immunized and unimmunized control birds to both *E. tenella* asexual stages, but previously, at 3 days of age, significant maternal antibodies transferred by passive immunization were detected by indirect ELISA. According to Wallach [20], passive immunity in birds is a major item to consider in the further development of their cell-mediated immunity against *Eimeria* species. Apparently, antibodies in naïve birds did not show any type of interference on the development of immunity against *E. tenella* sporozoites in the vaccinated group, and at 21 days of age, the immunized SPF chicks showed higher antibody titers than the naïve control birds. Yun et al. [18] argued that killed coccidia vaccines contain a wide array of parasite immunogens to induce humoral immunity; however, they usually lack critical components associated with the intracellular developmental stages necessary to activate cell-mediated immunity. Although chicks exposed to prolonged natural infection also develop antibodies to B antigens, the active immunization of young chicks with a subcutaneous dose of EtSz-IMS1313 might not elicit a significant mucosal antibody response to neutralize *Eimeria* infection, suggesting that the immune protection observed here could result from another cell-mediated effector mechanism. Therefore, further studies are needed to determine if a mucosal component might be involved to give rise to the immune protection observed here with the EtSz-IMS1313 vaccine.

Antibodies developed in rabbits and SPF birds immunized with whole sporozoites of *E. tenella* reacted similarly to the sporozoite and merozoite antigens, implying the presence of the same or cross-reacted antigens in both parasite stages, suggesting that most antigens are common to the two life cycle stages [25]. The increases in antibody levels against both stages were similar in magnitude, further supporting the argument that the two invasive stages share antigens. The antibodies from the SPF birds showed a higher level of OD units immediately after the first immunization, which helps to explain the successful results obtained in the immunization/challenge test. These results are in close conformity with those reported by Garg et al. [10], who recorded a significant increase in the antibody levels of chicks against the *E. tenella* sporozoite from 12 days PI.

In accordance with Talebi and Mulcahy [44], the increased titers of antibodies against *Eimeria* have a significant negative correlation with the oocyst output, Interestingly, we also observed an increase in antibody titers against *E. tenella* in our immunization/challenge trial. The antibody titers from rabbits increased at 14 days PI, peaked at 28 days, and then declined slightly to reach a plateau. This is consistent with previous reports where antibodies persist for prolonged periods in birds and rabbits that have received continuous booster doses [7,10,44,45]. The pattern of the antibody response indicates a maturation affinity event. It is indicative of effective acquired immunity against *E. tenella* that would occur immediately after second immunization [7,10,44,45].

The SDS-PAGE analysis of purified *E. tenella* sporozoites and the 2nd generation of merozoites showed that their polypeptide profiles are as complex as others previously reported [15,46,47,48]. In order to investigate a possible relationship between immunogenicity and the specific mass of proteins in the sporozoite and merozoite stages, immunoblots were performed with hyperimmune rabbit and chicken sera. Constantinoiu et al. [45,49], during their studies on the kinetics and avidity of antibodies against *Eimeria*, observed that antibodies against *E. tenella* were detected from the second week and peaked at 21 days PI; however, only after the third immunization did the antisera consistently identify more polypeptides in both *Eimeria* asexual stages.

In our immunoblotting study, there were some differences in the identification and staining intensities among antisera from rabbits and chickens at different times PI. Antibodies from the rabbits injected subcutaneously with the EtSz-IMS1313 vaccine exhibited the clearest immunogenic pattern in both asexual zoite stages immediately after first immunization. At least ten polypeptides were clearly recognized in the first analysis PI. The magnitude of the positive recognition of the antibodies was significantly increased immediately after the first booster dose; hence, every serum had to be progressively diluted. Western blot analysis allowed us to identify some antigenic differences between the sporozoites and merozoites, although the majority of the antigens seemed to be common between them. The sharing of antigens between sporozoites and merozoites has been demonstrated previously in studies with chicken and rabbit antisera [45,47,48,49]. As a matter of fact, most antigens with a molecular weight higher than 50 kDa seemed to be shared by these two life cycle stages [45,49].

Using information from all blots, it was possible to build a picture of the major determinants of antigenic cross-reactivity between the two developmental stages. In order to design an effective genetically engineered vaccine, the most promising antigens were derived from the early endogenous stages of the *Eimeria* life cycle (sporozoites and merozoites). This correlates with the findings observed in naturally infected chickens, where these stages induce the strongest anti-*Eimeria* immunity [8,45,47,48,49]. Many of these antigens have critical roles in host–parasite interactions [3,5,14,16]. As the compartmentalization of pathogen antigens is a major factor in their accessibility to host major histocompatibility complex (MHC), the proteins identified in apicomplexan parasites are regularly classified into secreted proteins, surface proteins, and internal proteins [2,16,21].

Some of these secreted proteins are trafficked to the parasite surface and beyond via the secretory microneme (MIC) organelles, such as AMA1, IMP1, MIC1, MIC2, MIC3, micronemal protein 4 (MIC4), and micronemal protein 5 (MIC5) [3,4,5,16,30,39,50]. Several of these proteins were identified in our blots based on their homologous protein mass. According to Kawazoe et al. [51], microneme epitopes are strongly conserved between sporozoites and 2nd generation merozoites, while the majority of rhoptry epitopes and many membrane antigens are sporozoite-specific. In our study, based on homologous molecular weight, some other proteins such as EtMIC3 (120 kDa), microneme protein precursor EtMIC1 (105 kDa), heat shock protein 70 (HSP70) (70 kDa), beta-tubulin (64 kDa), pyruvate kinase (58 kDa), glyceraldehyde 3-phosphate dehydrogenase (GAPDH) (55 kDa), EtMIC2 (53 kDa), aspartyl proteinase-eimepsine (44–47 kDa), 14-3-3 protein (38 kDa), surface antigen 12 (SAG12) (24 kDa), surface antigen 13 (SAG13) (23 kDa), homologous of 2-Cys peroxiredoxin Bas1 precursor (Prx) (*T. gondii*) (21 kDa), and profilin (19 kDa) were identified as common immunogenic antigens on sporozoites and 2nd generation merozoites of *E. tenella*, while enolase (50 kDa), EtMIC5 (44 kDa), and surface antigen 4 (SAG4) (19 kDa) were observed only from merozoite antigens [21,24,52].

In the present study, we identified three antigens (150, 94, and 12 kDa) without apparent homology to the *E. tenella* antigens previously described [21,24,52]. Compared to the sporozoite stage, Liu et al. [24] found that SAG, SAG2, SAG4, EtMIC5, SERPINI protein precursor, 14-3-3 protein, tubulin beta chain, large subunit ribosomal protein L23, and several homologous proteins to other apicomplexan parasites or protozoan were identified as stage-specific immunogenic proteins in the 2nd generation of merozoites. Nonetheless, some proteins, including EtMIC3, microneme protein precursor EtMIC-1, enolase, lactate dehydrogenase, and HSP70, were shown to be common antigens in the two invasion stages of sporozoites and the 2nd generation of merozoites. Recently, Liu et al. [21] determined that 14-3-3 and GAPDH are common antigens in *Eimeria* species. Hence, Et14-3-3 and EtGAPDH proteins might be potential vaccine candidates against cecal coccidiosis.

In the natural immune response of rabbits and birds to coccidia, the sporozoite surface and secreted proteins could be directly recognized by the host immune system and subsequently induce a host immune response [2,8,44,48]. In contrast, the internal proteins are usually not recognized by the host immune system and do not induce antibody responses in the host [18,53]. However, in our study, eight internal proteins seem to have been recognized by both animal antisera. This indicates that these proteins were processed by the host. The reasons why these internal proteins could be recognized by the host immune system perhaps relate to two facts: these proteins were secretory, or they were released by antigen-presenting cells (ACPs) and subsequently processed by the host immune system. The proteins and surface antigens of *E. tenella* secreted at the sporozoite stage play an essential role in the host–parasite interaction, which involves attachment and invasion. Therefore, these interacting proteins should be considered as vaccine candidates based on the strategy of cutting off the invasion pathway to interrupt infection. The successful identification and testing of these proteins will expedite the development of a genetically engineering vaccine capable of inducing adequate protection against this parasite. Further studies to evaluate the combination of whole sporozoites from other *Eimeria* species emulsified in nanoparticles are currently being evaluated.

## 5. Conclusions

The immunogen based on whole sporozoites of *E. tenella* emulsified in the nanoparticle adjuvant IMS 1313 N VG (Montanide™) generate a successful immune protection against challenge with a heavy dose of *E. tenella*, showing its potential for use as a vaccine. This vaccine allowed the identification of several B-cell epitopes implicated in the protective immunity against *E. tenella.*

## Figures and Tables

**Table 1:** Effects of *E. tenella* whole sporozoite vaccine immunization on the 1st and 10th days of age against a challenge at 21st days of age with 30,000 sporulated oocysts of a wild-type *E. tenella* strain on the parameters of immune protection.

Groups	Survival Rate (%)	Average Body Weight Gain (g)	Relative Body Weight Gain Rate (%)	Mean Lesion Scores (Mean ± SD)
Unchallenged control	100.0 ^a^	63.07 ± 7.83 ^a^	100.0	0.00 ± 0.00 ^c^
Vaccinated group	100.0 ^a^	59.79 ± 5.33 ^a^	94.8	0.69 ± 0.70 ^c^
Adjuvant group	71.0 ^b^	14.52 ± 2.06 ^b^	23.0	3.03 ± 0.79 ^b^
Challenge control	76.9 ^b^	−17.14 ± 3.80 ^c^	−27.2	3.80 ± 0.25 ^a^
	**Lesion Score Protective Index (%)**	**Oocyst Output (× 10^6^) (Mean ± SD)**	**Oocysts Output Decrease Ratio (%)**	**Anticoccidial Index**
Unchallenged control	100.00	0.00 ± 0.00 ^c^	100.00	200
Vaccinated group	81.78	0.14 ± 0.09 ^b^	95.8	186
Adjuvant group	20.34	3.93 ± 0.36 ^a^	−14.3	18
Challenge control	0.00	3.44 ± 1.22 ^a^	0.00	−28

Note: In every column, there was a significant difference (*p* < 0.05) between numbers with different letters. No significant difference was shown between values with the same letter.

## Data Availability

The datasets generated for this study are available on request to the corresponding author.
